# Distinct care trajectories among persons living with arthritic conditions: A two-year state sequence analysis

**DOI:** 10.3389/fpain.2022.1014793

**Published:** 2022-11-12

**Authors:** Hermine Lore Nguena Nguefack, M. Gabrielle Pagé, Manon Choinière, Alain Vanasse, Simon Deslauriers, Adriana Angarita-Fonseca, Marc-André Blanchette, Anaïs Lacasse

**Affiliations:** ^1^ Department of Health Sciences, Université du Québec en Abitibi-Témiscamingue, Rouyn-Noranda, QC, Canada; ^2^ Research Centre, Centre Hospitalier de l’Université de Montréal, Montreal, QC, Canada; ^3^ Department of Anesthesiology and Pain Medicine, Faculty of Medicine, Université de Montréal, Montreal, QC, Canada; ^4^ Department of Family Medicine and Emergency Medicine, Faculty of Medicine and Health Sciences, Université de Sherbrooke, Sherbrooke, QC, Canada; ^5^ Research Centre, Centre Hospitalier Universitaire de Sherbrooke, Sherbrooke, QC, Canada; ^6^ VITAM – Centre de recherche en santé durable, Centre Intégré Universitaire de Santé et de Services Sociaux de la Capitale-Nationale, Quebec, QC, Canada; ^7^ Département de Chiropratique, Université du Québec à Trois-Rivières, Trois-Rivières, QC, Canada

**Keywords:** state sequence analysis, cluster, arthritis, care trajectories, pain, health care utilization, Canadian community health survey

## Abstract

**Objectives:**

Developing solutions to optimize care trajectories (CareTs) requires examining patient journeys through the health care system. This study aimed to describe CareTs among people living with arthritis and evaluate their association with self-reported health outcomes.

**Methods:**

Analyses were conducted using the TorSaDE Cohort (*n* = 102,148), which connects the 2007 to 2016 Canadian Community Health Surveys (CCHS) with Quebec administrative databases (longitudinal claims). CareTs of participants living with arthritis according to CCHS (*n* = 16,631), over the two years before CCHS completion, were clustered using state sequence analysis (months as a time unit). CareT group membership was then put in association with self-reported outcomes (pain intensity and interference, self-perceived general and mental health).

**Results:**

The analysis revealed five CareT groups characterized predominantly by: (1) arthritis-related visits to a specialist (*n* = 2,756; 16.6%), (2) arthritis-related emergency department visits (*n* = 2,928; 17.6%), (3) very high all-cause health care utilization and arthritis-related hospitalizations (*n* = 1,570; 9.4%), (4) arthritis-related medical visits to general practitioners and specialists (*n* = 2,708; 16.3%), (5) low all-cause health care utilization (*n* = 6,669; 40.1%). Multivariable results revealed that CareT group membership was associated with higher levels of pain interference (CareT group #3 vs. #5: OR: 1.4, 95%CI: 1.1–1.8) and fair/poor self-perceived general health (CareT group #1 vs. #5: OR: 1.551, 95%CI: 1.319–1.824; #2 vs. #5: OR: 1.244, 95%CI: 1.062–1.457; #3 vs. #5: OR: 1.771, 95%CI: 1.451–2.162; #4 vs. #5: OR: 1.481, 95%CI: 1.265–1.735).

**Discussion:**

Sate sequence analysis is an innovative method of studying CareTs and valuable for making evidence-based decisions taking into account inter- and intra-individual variability.

## Introduction

Arthritis includes several conditions that affect joints, the tissues surrounding joints, and other connective tissues (1). In Canada, arthritis is the most prevalent chronic disease, carrying a significant financial burden due to loss of productivity, disability, and increased health care costs (1, 2). This health condition affects approximately 20% of Canadians (2, 3). Over the next decade, an increase of about 3 million cases is expected, which will translate into 9 million persons living with arthritis (3). A number of negative physical, mental, and social consequences have been demonstrated as a result of arthritis (1, 3), which often translate into ambulatory care visits (1).

According to the “6W” model, individuals (who) living with various health conditions (why) will consult various types of health care professionals (which) in different care settings (where). Patients will receive a sequence of services (what) over specific periods of time (when) (4). Care trajectories can thus be defined as “*patients' journeys through different components of the health care system over their chronic illness courses, care providers, care units and settings, and over time*” (4). Understanding the care trajectories (CareTs) of people living with arthritis can provide policymakers and clinicians with valuable insight for improving the organization of health care and determining optimal planning of care processes (5). However, few studies have investigated CareTs in people living with arthritis. Ruetsch et al. (6) identified four CareT groups among people living with low back pain or osteoarthritis. Group membership was carried out by k-means clustering, where cases were separated into high and low service use intensity for each year using US claim data. However, this technique failed to account for the longitudinal nature of the data (6). Kiadaliri et al. (7) identified four trajectories of physical health care consultations (total number of primary care visits, secondary outpatient care visits, and hospital admissions) within a Swedish osteoarthritis cohort, using group-based trajectory modelling. Moreover, Mose et al. (8) found five 10-year trajectories of musculoskeletal health care use (total number of various types of health care contacts) among adult Danes reporting chronic musculoskeletal pain. The Kiadaliri's and Mose's studies (7, 8) did model the number of health care contacts over time but did not consider which event occurred before another in time (sequence of events). However, this is important to realize that hierarchy and continuity of care can influence the quality of care and patient outcomes (9).

The objective of this study was to describe the heterogeneity of longitudinal CareTs among people living with arthritis using state sequence analysis, an emerging statistical method allowing to group participants showing similar patterns of health care visits over time. The sociodemographic and clinical profile of the individuals who were grouped together in each of the CareT was then compared. Finally, the associations between CareT membership and patient-reported outcomes such as pain intensity, pain interference, perceived general health and perceived mental health were assessed.

## Methods

### Study design and data source

This observational retrospective cohort study was conducted using the TorSaDE Cohort (10), which was created by linking data from Quebec health administrative databases and the Canadian Community Health Survey (CCHS) database. The CCHS, an annual cross-sectional survey conducted by Statistics Canada (national statistical office), collects data on health status and health care utilization using a representative sample of the Canadian population (11). The TorSaDE Cohort includes CCHS respondents from the province of Quebec who participated in at least one of the five annual cycles (2007–2008, 2009–2010, 2011–2012, 2013–2014, 2015–2016). Quebec health administrative databases were used, which included information about health care services, hospitalizations, diagnoses, and health insurance registration provided to all Quebec residents (universal health care coverage). The TorSaDE Cohort contains longitudinal administrative data from 1996 to 2016. A detailed description of the TorSaDE Cohort is available elsewhere (10). Access to de-identified TorSaDE Cohort data was possible through the *Institut de la statistique du Québec* (ISQ) secure virtual server (data holder). Ethical authorizations were obtained from the *Commission d'accès à l'information du Québec* (#1013990) and relevant university Research Ethics Boards (UQAT: # 2018–02 –Lacasse, A.; CHUS: #2017–1504).

### Study population

**Figure 1:**
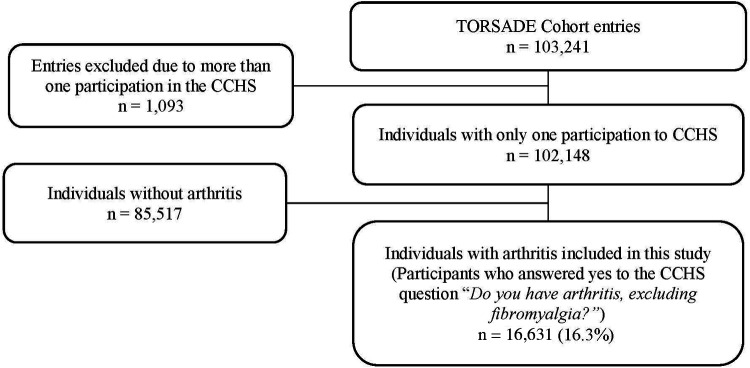
Study sample selection.

The TorSaDE Cohort includes 102,148 individual participants who completed 103,241 entries (individuals can take part in more than one CCHS cycle). As shown in Figure 1, those who met the following inclusion criteria formed our study population: (1) for individuals with more than one CCHS entry, only the most recent entry was retained (*n* = 102,148); and (2) participants who responded “yes” to the CCHS question “Do you have arthritis, excluding fibromyalgia?” (*n* = 16,631). Although arthritis could have been defined using case-finding algorithms applied to administrative health claims (12–15), preference was given to self-reported CCHS data as it allowed for the formation of a community sample of people living with arthritis that includes individuals with little or no contact with the health care system and who are often understudied in administrative database health service research studies. The reliability of self-reported chronic conditions in the CCHS have been shown to be high (16).

### Study variables

***Care trajectories.*** CareTs in the two years before CCHS completion were modelled using health administrative data (time unit: months). State sequence analysis based on optimal matching was conducted and allowed to cluster individuals with similar patterns of health care visits over time into various CareT groups (see complete description of the statistical analysis). CareT group membership was then put in association with various patient-reported outcomes measured in the CCHS. ***Outcomes of interest.*** Four CCHS outcomes relevant to pain and health-related quality of life were chosen and put in association with CareTs. The outcomes were as follows: (**1**) pain intensity [respondents reporting pain or discomfort were asked “*How would you describe the usual intensity of your pain or discomfort*?” (Mild/Moderate/Severe)]; when respondents reported no pain/discomfort, pain intensity was coded as “None”); (**2**) pain interference [“*How many activities does your pain or discomfort prevent*?” (None/A few/Some/Most)]; (**3**) perceived general health [“*In general, would you say your health is…?*” (Excellent/Very good/Good/Fair/Poor)]; (**4**) perceived mental health [“*In general, would you say your mental health is…?*” (Excellent/Very good/Good/Fair/Poor)]. The reliability of responses to CCHS questions has been previously demonstrated (16). ***Covariables***. Covariables were selected according to Andersen's (1995) model (17), which is widely used in health care utilization studies (18). Factors put forward in this model include: (**1**) health care utilization predisposing factors, i.e., in our study: biological sex, age, race/ethnicity, indigenous identity, country of birth, education, marital status, alcohol consumption, and smoking habits, (**2**) enabling factors: region of residence (remote, non-remote, rural, urban), income, drug insurance status, access to a general practitioner, number of different prescribers, and access to a pain clinic, and (**3**) need factors: back problems, Charlson Comorbidity Index (19, 20), and body mass index. To be selected, covariables had to be measured in the five CCHS cycles included in the TorSaDE Cohort and had to be available for at least 15% of our sample (for example, work-related variables were only measured among workers aged 18 to 50 years; other variables are not measured in all CCHS cycle and were thus excluded).

### Statistical analysis

Our statistical plan was developed in other to reflect as best as possible the components of the “6W” model (4). ***Study population characteristics.*** Descriptive statistics (means, standard deviations, counts and percentages) were used to summarize the main characteristics of the study sample. ***State sequence analysis.*** Identification of CareT group membership [i.e., individuals following similar journeys through different components of the health care system over their chronic illness courses, care providers, care units and settings, and over time” (4)] was completed using state sequence analysis (21, 22). The complete description of the state sequence analysis, including (1) Defining the cohort of patients, the observation period, and the time unit, (2) Choice and prioritization of medical events (states) of interest, (3) Use of an appropriate distance or similarity measure method to calculate the distance between the pairs of sequences, resulting in a distance matrix, (4) Selection and application of a classification method based on the calculated distance matrix that results in distinct groups of patients sharing similar patterns of health care utilization (CareT groups), and (5) visual representation of CareT using chronograms (state distribution plots), is presented in Supplementary Digital Content S1. ***Profiles of the various CareT groups.*** In addition to chronograms and narrative descriptions of each CareT group, descriptive statistics were also computed (membership count and sociodemographic/clinical profile). ***CareT groups in relation to self-reported outcomes.*** Pain intensity, pain interference, perceived general health, and perceived mental health were first compared across CareT groups using bivariate analyses (Chi-square and Kruskal-Wallis tests). Post-hoc pairwise analysis with *p*-value corrections were performed using Tukey-style multiple comparisons of proportions and Dwass-Steel-Critchlow-Fligner tests. Multivariable logistic regression models were used to assess the association between CareT group membership (independent variable) and two of our outcomes of interest while accounting for confounding factors (for a matter of conciseness, only pain interference and perceived general health were modelled using multivariate analyses). Those two outcomes were prioritized because they covered both pain-specific and generic aspects of patients daily living. In the two multivariable logistic regression models aiming at predicting unfavourable outcomes, pain interference response categories were dichotomized as: 1 = Most/some activities prevented by pain or discomfort; 0 = None/A few activities prevented by pain or discomfort. Perceived general health response categories were dichotomized as: 1 = Poor/Fair; 0 = Excellent/Very good/Good. Odds ratios (OR) and 95% confidence intervals (CI) were calculated. CareT group #5 (“low utilization”) was chosen as the reference category for interpretation. As per recent literature recommendations, all covariables were identified *a priori* and included in the model (as opposed to other criticized covariate selection techniques such as relying on bivariate regression analysis *p*-values (23) or stepwise variable selection (24, 25)). Variance inflation factors (VIFs) below 5 (26) were used in order to detect variables with a multicollinearity issue. Various statistics such as the Hosmer-Lemeshow test (values for model 1 and model 2: *p* = 0.2598 and *p* < .0001) and the c index (0.889 and 0.832) supported the quality of our models. A sensitivity analysis was carried out to assess the impact of a multiple imputation technique for missing data (25) on our conclusions. Multiple imputation by fully conditional specification (FCS) method was applied with 5 repetitions. State sequence analysis was performed using the R package TraMineR (R Core Team, Vienna, Austria) while all other analyses were carried out with SAS 9.4 (SAS Institute, Cary, NC, USA).

## Results

### Sample characteristics

The characteristics of study participants are shown in Table 1 (*n* = 16,631). Mean age was 65.5 ± 13.5 years, 66.7% of participants were females and 2.6% self-identified as Indigenous people. Only half of participants (52.6%) had a medical claim associated with an arthritic condition diagnosis in the two years prior to CCHS completion (non mutually exclusive variables: 28.0% with osteoarthritis diagnosis, 5.7% with a rheumatoid arthritis diagnosis, 37.2% with other arthritic conditions diagnosis). A third (32.3%) perceived their general health as poor or fair, while 33.8% did so for their mental health.

**Table 1:** Sample characteristics.

Characteristics^a^ (*n* = 16 631)	No. (%) of participants^b^	
**Sociodemographic profile**		
**Age (years)**—mean ± SD	65.55	±13.50
**Biological sex**		
Females	11,088	(66.67)
Males	5,543	(33.33)
**Caucasian self-identification**		
Yes	15,783	(94.90)
No^c^	848	(5.10)
**Indigenous self-identification** ^d^		
Yes	410	(2.58)
No	15,479	(97.42)
**Country of birth**		
Canada	15,522	(93.33)
Other	1,109	(6.67)
**Education level**		
No high school diploma	6,147	(37.60)
High school diploma	2,455	(15.02)
Post-secondary diploma	5,323	(32.56)
University diploma	2,423	(14.82)
**Marital status**		
In a relationship	8,183	(49.22)
Not in a relationship	8,441	(50.78)
**Household income (CAN$)**		
<20,000	3,918	(23.56)
20,000–39,999	5,476	(32.93)
40,000–59,999	3,198	(19.23)
60,000–79,999	1,802	(10.84)
≥80,000	2,237	(13.45)
**Region of residence** ^e^		
Remote region	4,124	(24.80)
Non-remote region	12,507	(75.20)
**Geographic area** ^f^		
Urban	11,805	(70.98)
Rural	4,826	(29.02)
**Drug insurance status (in the 2 years prior to CCHS completion)**		
Public	11,363	(68.32)
Private	5,268	(31.68)
**Pain symptoms**		
**Back pain, except fibromyalgia and arthritis**		
Yes	5,766	(34.80)
No	10,801	(65.20)
**Arthritis conditions (claims in the 2 years prior to CCHS completion)**		
**Rheumatoid arthritis**		
Yes	935	(5.68)
No	15,516	(94.32)
**Osteoarthritis**		
Yes	4,609	(28.02)
No	11,842	(71.98)
**Other arthritis conditions** ^g^		
Yes	6,114	(37.16)
No	10,337	(62.84)
**At least one above-mentioned arthritis conditions**		
Yes	8,659	(52.64)
No	7,792	(47.36)
**General health profile**		
**Charlson comorbidity index**– mean ± SD	0.59	±1.60

SD: Standard deviation.

^a^ Proportion of missing data across presented variable ranges between 0 and 4.46%.

^b^ Unless stated otherwise.

^c^ South Asian (e.g., East Indian, Pakistani, Sri Lankan), Chinese, Black, Filipino, Latin American, Arab, Southeast Asian (e.g., Vietnamese, Cambodian, Malaysian, Laotian), West Asian (e.g., Iranian, Afghan), Korean, Japanese, Other.

^d^ CCHS do not cover Indigenous people living on-reserve.

^e^ Remote resource regions as defined by Revenu Quebec (i.e., the provincial revenue agency): Bas-Saint-Laurent (region 01), Saguenay–Lac-Saint-Jean (region 02), Abitibi-Témiscamingue (region 08), Côte-Nord (region 09), Nord-du-Québec (region 10), Gaspésie–Îles-de-la-Madeleine (region 11). Nonremote regions are near a major urban center.

^f^ A Canadian postal code with a zero “0” in the second position denotes “rural”.

^g^ Gout, polymyalgia rheumatic, ankylosing spondylitis, other seronegative spondyloarthropathy, psoriasis, synovitis/tenosynovitis/bursitis, other disorders of synovium tendon and bursa, connective tissue disorder, other disorders of soft tissues, vasculitis, Ooher and unspecified arthropathies, disorders of muscle ligament and fascia, symptoms involving nervous and musculoskeletal systems, other derangement of joint.

### Narrative description of CareT groups

**Figure 2:**
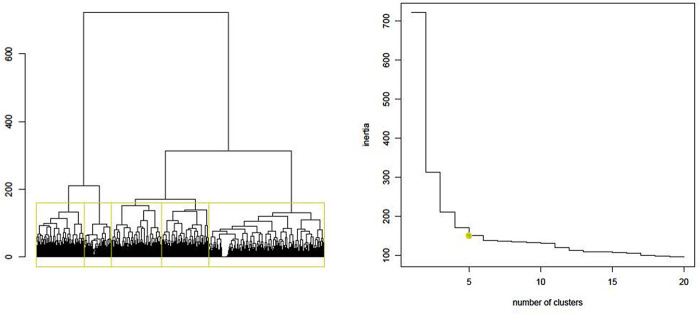
Dendrogram and inertia jump curve for the choice of the number of groups.

**Figure 3:**
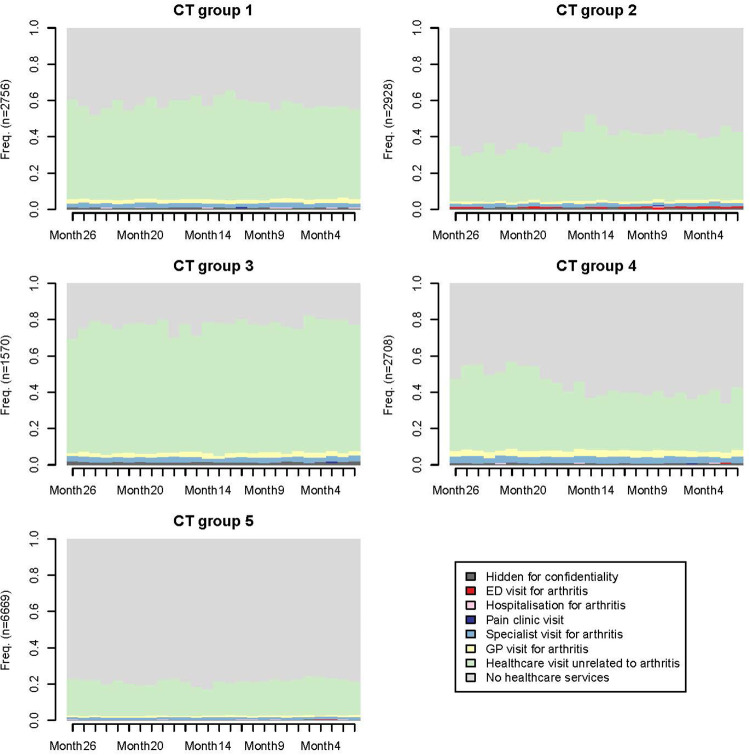
Chronograms reflecting patterns of health care visits over time for each CareT group.

Based on the state sequence analysis dendrogram (represents similarities between participants and how they can be grouped) and inertia jump curve (from five, we see that the increase in the number of trajectories/clusters does not provide much more information, i.e., inertia) (Figure 2), patients with similar care sequences were classified into five distinct CareT groups (Figure 3 where each colour represents frequency of different medical events throughout time). According to chronograms' interpretation, their particularities in terms of all-cause health care utilization and arthritis-related visits were described and are presented in Table 2. CareT group #1 was made up of participants with high all-cause health care utilization and frequent arthritis-related ambulatory medical visits with predominance of visits to a specialist as compared to other groups (“high utilization/specialists” *n* = 2,756; 16.6%). CareT group #2 consisted of participants with moderate all-cause health care utilization and more frequent arthritis-related ED visits than the other groups (“moderate utilization/ED” *n* = 2,928; 17.6%). CareT group #3 included participants with very high all-cause health care utilization and more frequent arthritis-related hospitalizations than the other groups (“very high utilization/arthritis-related hospitalizations” *n* = 1,570; 9.4%). CareT group #4 was made up of participants with moderate all-cause health care utilization and more frequent arthritis-related medical visits to GPs or specialists than the other groups (“moderate utilization/arthritis-related physicians” *n* = 2,708; 16.3%). Lastly, CareT group #5 was made up of participants with low all-cause health care utilization (“low utilization” *n* = 6,669; 40.1%).

**Table 2:** CareT groups’ description.

Care trajectory (CareT) group	All causes number of contacts with the health care system	Arthritis-related visits	Where this group stands out in terms of type of arthritis -related visits (as compared to other groups)
1	High	Moderate use (relatively similar across these three groups)	Visits to specialists
2	Moderate		Emergency department visits
3	Very high		Hospitalizations
4	Moderate	Slightly greater than the previous groups	Visits to general practitioners and other specialists
5	Low	Low	NA

NA, not available.

### CareT group profiles

Based on bivariate statistical comparisons (without taking confounding factors into account), CareT groups differed in terms of sociodemographic characteristics, utilization of health care services during the two years prior to CCHS completion (Table 3), and health outcomes (Table 4). Specifically, participants in the “very high utilization/arthritis-related hospitalizations” group showed the highest proportion of females (71.2%), participants with no high school diploma (43.3%), participants with an annual household income under CAN$20,000 (31.2%), and individuals with medical claims related to osteoarthritis (35.7%). Furthermore, it featured the smallest proportion of participants residing in a remote region (18.66%). In addition, participants in that group had the highest average number of all-cause GP ambulatory visits (7.00 ± 5.54), specialist ambulatory visits (11.03 ± 14.97), hospitalizations (0.58 ± 0.94), and ED visits (1.42 ± 2.12) in the two years prior to CCHS completion. The “very high utilization/arthritis-related hospitalizations” group also had the highest average Charlson Comorbidity Index (1.85 ± 2.73). The “low utilization” group had the highest proportion of males, participants residing in a remote region and individuals with higher socioeconomic status.

**Table 3:** Arthritis patients’ characteristics according to their CareT group membership.

Population characteristics	#1. High utilization/specialists 2,756 (16.57)	#2. Moderate utilization/ED 2,928 (17.61)	#3. Very high utilization/ arthritis-related hospitalizations 1,570 (9.44)	#4. Moderate utilization/arthritis-related physicians 2,708 (16.28)	#5. Low utilization 6,669 (40.10)	*p*-value^a^
**Age**—mean ± SD	67.89 ± 12.91	66.08 ± 13.21	68.71 ± 12.99	66.47 ± 12.95	63.23 ± 13.82	**<.0001** Post-hoc differences: 1–2, 1–4, 1–5, 2–3, 2–5, 3–4, 3–5, 4–5
**Sex**—***n* (%)**						**<.0001** Post-hoc differences: 1–5, 2–5, 3–5, 4–5
Females	1,906 (69.16)	2,014 (68.78)	1,117 (71.15)	1,909 (70.49)	4,142 (62.11)	
Males	850 (30.84)	914 (31.22)	453 (28.85)	799 (29.51)	2,527 (37.89)	
**Education level—*n* (%)**						**<.0001**
No high school diploma	1,075 (39.77)	1,075 (37.35)	667 (43.26)	1,038 (38.99)	2,292 (34.92)	
High school diploma	390 (14.43)	460 (15.98)	214 (13.88)	396 (14.88)	995 (15.16)	
Post-secondary diploma	867 (32.08)	894 (31.06)	474 (30.74)	853 (32.04)	2,235 (34.05)	
University diploma	371 (13.73)	449 (15.60)	187 (12.13)	375 (14.09)	1,041 (15.86)	
**Household income (CAN$)—*n* (%)**						**<.0001**
<20 000	733 (26.60)	691 (23.60)	489 (31.15)	605 (22.34)	1,400 (20.99)	
20 000–39 999	950 (34.47)	984 (33.61)	560 (35.67)	942 (34.79)	2,040 (30.59)	
40 000–59 999	513 (18.61)	561 (19.16)	273 (17.39)	541 (19.98)	1,310 (19.64)	
60 000–79 999	267 (9.69)	311 (10.62)	125 (7.96)	288 (10.64)	811 (12.16)	
≥80 000	293 (10.63)	381 (13.01)	123 (7.83)	332 (12.26)	1,108 (16.61)	
**Region of residence—*n* (%)**						**<.0001** Post-hoc differences: 1–5, 2–5, 3–5, 4–5, 1–2, 3–2, 4–3
Remote region	597 (21.66)	735 (25.10)	293 (18.66)	645 (23.82)	1,854 (27.80)	
Non-remote region	2,159 (78.34)	2,193 (74.90)	1,277 (81.34)	2,063 (76.18)	4,815 (72.20)	
**Access to a pain clinic– *n* (%)**						**<.0001** Post-hoc differences: 1–5, 2–5, 3–5, 4–5
Yes	190 (6.89)	212 (7.24)	110 (7.01)	172 (6.35)	310 (4.66)	
No	2,566 (93.11)	2,716 (92.76)	1,460 (92.99)	2,536 (93.65)	6,344 (95.34)	
**Rheumatoid arthritis medical claims**—***n* (%)**						**<.0001** Post-hoc differences: 1–5, 2–5, 3–5, 4–5, 1–4, 2–4, 3–4
Yes	190 (6.89)	155 (5.30)	92 (5.86)	245 (9.05)	253 (3.90)	
No	2,566 (93.11)	2,772 (94.70)	1,478 (94.14)	2,462 (90.95)	6,238 (96.10)	
**Osteoarthritis medical claims**—***n* (%)**						**<.0001** Post-hoc differences: 1–5, 2–5, 3–5, 4–5, 1–2, 3–2, 4–2
Yes	945 (34.29)	865 (29.55)	561 (35.73)	889 (32.84)	1,349 (20.78)	
No	1,811 (65.71)	2,062 (70.45)	1,009 (64.27)	1,818 (67.16)	5,142 (79.22)	
**Medical claims related to other arthritis conditions except Rheumatoid arthritis and Osteoarthritis– *n* (%)**						**<.0001** Post-hoc differences: 1–2, 1–3, 1–5, 2–3, 2–4, 2–5, 3–4, 3–5, 4–5
Yes	1,241 (45.03)	1,097 (37.48)	855 (54.46)	1,170 (43.22)	1,751 (26.98)	
No	1,515 (54.97)	1,830 (62.52)	715 (45.54)	1,537 (56.78)	4,740 (73.02)	
**Charlson comorbidity index**– mean ± SD	1.03 ± 2.04	0.51 ± 1.44	1.85 ± 2.73	0.48 ± 1.32	0.18 ± 0.83	**<.0001**
**Number of GP ambulatory visits (all cause)—**mean ± SD	4.90 ± 3.34	3.03 ± 2.17	7.00 ± 5.54	4.03 ± 3.07	1.71 ± 1.68	**<.0001** Post-hoc differences: 1–2, 1–3, 1–4, 1–5, 2–3, 2–4, 2–5, 3–4, 3–5, 4–5
**Number of specialists ambulatory (all cause) visits**—mean ± SD	5.95 ± 5.63	2.73 ± 2.88	11.03 ± 14.97	4.38 ± 4.29	1.32 ± 1.87	**<.0001** Post-hoc differences: 1–2, 1–3, 1–4, 1–5, 2–3, 2–4, 2–5, 3–4, 3–5, 4–5
**Number of episode of hospitalizations (all cause)**—mean ± SD	0.39 ± 0.76	0.17 ± 0.49	0.58 ± 0.94	0.30 ± 0.63	0.09 ± 0.34	**<.0001** Post-hoc differences: 1–2, 1–3, 1–4, 1–5, 2–3, 2–4, 2–5, 3–4, 3–5, 4–5
**Number of ED visits (all cause)**—mean ± SD	0.91 ± 1.61	0.48 ± 0.97	1.42 ± 2.12	0.70 ± 1.34	0.29 ± 0.71	**<.0001** Post-hoc differences: 1–2, 1–3, 1–4, 1–5, 2–3, 2–4, 2–5, 3–4, 3–5, 4–5

*P*-values < .05 are reported in bold.

SD: Standard deviation.

^a^ Chi-square tests or Kruskal-Wallis tests with Tukey style multiple comparisons of proportions or Dwass-Steel-Critchlow-Fligner tests for post-hoc pairwise analyses.

Proportion of missing data across presented variable ranges between 0% and 4.46%.

**Table 4:** Health outcomes of interest according to CareT group membership.

Health outcomes of interest and population characteristics	#1. High utilization/specialists 2,756 (16.57)	#2. Moderate utilization/ED 2,928 (17.61)	#3. Very high utilization/ arthritis-related hospitalizations 1,570 (9.44)	#4. Moderate utilization/arthritis-related physicians 2,708 (16.28)	#5. Low utilization 6,669 (40.10)	*p*-value^a^
**Pain Intensity—*n* (%)**						**<.0001** Post-hoc differences: 1–2, 1–3, 1–4, 1–5, 2–3, 2–5, 3–4, 3–5, 4–5
None/Mild	979 (50.08)	1 143 (56.58)	515 (43.98)	1 043 (54.15)	2 900 (63.24)	
Moderate/Severe	976 (49.92)	877 (43.42)	656 (56.02)	883 (45.85)	1 686 (36.76)	
**Activities prevented by pain or discomfort—*n* (%)**						**<.0001** Post-hoc differences: 1–2, 1–3, 1–4, 1–5, 2–3, 2–5, 3–4, 3–5, 4–5
None/None/A few	1 528 (77.88)	1 680 (82.92)	809 (69.09)	1 569 (81.51)	3 983 (86.76)	
Some/Most	434 (22.12)	346 (17.08)	362 (30.91)	356 (18.49)	608 (13.24)	
**Self-perceived general health—*n* (%)**						**<.0001** Post-hoc differences: 1–2, 1–3, 1–4, 1–5, 2–3, 2–5, 3–4, 3–5, 4–5
Excellent/Very good/Good	1,753 (63.68)	2,137 (73.03)	774 (49.43)	1,896 (70.17)	5,332 (80.05)	
Fair/Poor	1,000 (36.32)	789 (26.97)	792 (50.57)	806 (29.83)	1,329 (19.95)	
**Self-perceived mental health—*n* (%)**						**<.0001** Post-hoc differences: 3–1, 3–2, 3–4, 3–5, 5–1, 5–4
Excellent/Very good/Good	2,419 (91.63)	2,654 (93.42)	1,316 (87.56)	2,420 (92.65)	6,127 (94.51)	
Fair/Poor	221 (8.37)	187 (6.58)	187 (12.44)	192 (7.35)	356 (5.49)	

Proportion of missing data across presented variable ranges between 0.14% and 29.90%. *p*-values < .05 are reported in bold.

^a^ Chi-square test with Tukey style multiple comparisons of proportions test for post-hoc pairwise analyses.

Regarding self-reported health outcomes (Table 4), participants in the “very high utilization/arthritis-related hospitalizations” group had a higher proportion of moderate to severe pain intensity (56.02%), some or most activities prevented by pain or discomfort (30.91%), fair/poor self-perceived general health (50.57%), and fair/poor self-perceived mental health (12.44%) as compared to participants in the other CareT groups. The “low utilization” group showed the best outcomes compared to the other groups.

### Multivariable analysis of pain interference

The main results of the multivariable logistic regression model used to assess the association between CareT group membership and pain interference while adjusting for sociodemographic and clinical characteristics are shown in Table 5. Analysis revealed that membership to the “very high utilization/arthritis-related hospitalizations” group was predictive of greater pain interference (associated with a greater risk of seeing some or most of the activities prevented by pain or discomfort) as compared to participants in the “low utilization” group (adjusted OR: 1.4, 95% CI: 1.1–1.8). The complete results of the regression model (including coefficients for all other covariables associated with pain interference) are presented in Supplementary Digital Content S2.

**Table 5:** Multivariable logistic regression model exploring associations between CareT groups membership and greater pain interference (some/most activities prevented by pain or discomfort).

CareT groups membership (vs. Low utilization)	Adjusted OR^a^	95% Confidence interval	*p*-value	
High utilization/specialists	1.138	0.934	1.388	0.2001
Moderate utilization/ED	0.978	0.804	1.191	0.8272
Very high utilization/arthritis-related hospitalizations	**1**.**432**	**1**.**132**	**1**.**811**	**0**.**0027**
Moderate utilization/arthritis-related physicians	0.999	0.820	1.216	0.9919

*P*-values are reported in bold.

^a^ Adjusted For age, sex, race/ethnicity, indigenous, country of birth, education level, marital status, household income, region of residence, geographic area, CCHS cycle, public drug insurance status, access to a pain clinic, access to a general practitioner, number of different prescribers in the past 12 months, rheumatoid arthritis medical claims, osteoarthritis medical claims, medical claims related to other arthritis conditions except rheumatoid arthritis and osteoarthritis, pain intensity, back problems excluding fibromyalgia and arthritis, Charlson comorbidity index in the past 12 months, self-perceived general health, self-perceived mental health, kind of alcohol consumption in the past 12 months, kind of smoker, body mass index; 9,989 participants with no missing data were included in the final model.

### Multivariable analysis of self-perceived general health

Table 6 provides the main findings of the multivariable model used to evaluate the association between CareT group membership and self-perceived general health. Compared to the participants in the “low utilization” group, membership to all other CareT groups was associated with a higher likelihood of perceiving their general health as poor or fair (“high utilization/specialists”: aOR: 1.6, 95% CI: 1.3–1.8; “moderate utilization/ED”: aOR: 1.2, 95% CI: 1.1–1.5; “very high utilization/arthritis-related hospitalizations”: aOR: 1.8, 95% CI: 1.5–2.2; “moderate utilization/arthritis-related physicians”: aOR: 1.5, 95% CI: 1.3–1.7). The complete results of the multivariable model are presented in Supplementary Digital Content S3.

**Table 6:** Multivariable logistic regression model exploring associations between CareT groups membership and poor/fair self-perceived general health.

CareT groups membership (vs. Low utilization)	Adjusted OR^a^	95% Confidence interval		*p*-value
High utilization/specialists	1.553	1.321	1.826	<.0001
Moderate utilization/ED	1.247	1.064	1.460	0.0063
Very high utilization/arthritis-related hospitalizations	1.783	1.461	2.177	<.0001
Moderate utilization/arthritis-related physicians	1.483	1.266	1.737	<.0001

*P*-values are reported in bold.

^a^ Adjusted For age, sex, race/ethnicity, indigenous, country of birth, education level, marital status, household income, region of residence, geographic area, CCHS cycle, public drug insurance status, access to a pain clinic, access to a general practitioner, number of different prescribers in the past 12 months, rheumatoid arthritis medical claims, osteoarthritis medical claims, medical claims related to other arthritis conditions except rheumatoid arthritis and osteoarthritis, pain intensity, pain interference, back problems excluding fibromyalgia and arthritis, Charlson comorbidity index in the past 12 months, self-perceived mental health, kind of alcohol consumption in the past 12 months, kind of smoker, body mass index; 9,989 participants with no missing data were included in the final model.

Multiple imputation of missing values did not change our main conclusions of the two above-mentioned multivariable analyses (the models are presented in Supplementary Digital Contents S4 and S5).

## Discussion

This study aimed to identify CareT among 16,631 people living with arthritis and to explore the associations between CareT group membership and patient-reported outcomes such as pain intensity, pain interference, perceived general health and perceived mental health. State sequence analysis allowed for the identification of five CareT groups that were heterogenous in terms of all-cause health care utilization and arthritis-related visits patterns. Patient-reported outcomes and sociodemographic profiles varied according to CareT trajectory membership.

Several authors studied health care utilization and journeys through the health care system of persons living with arthritis in Canada (5, 27–30). However, individual-centered statistical approaches (22) were rarely used to better capture inter- and intra-individual variability of CareTs in this population. On an international scale, no study to our knowledge has looked at the heterogeneity of CareT profile among persons living with arthritis using state sequence analysis, but some comparisons are possible with longitudinal studies conducted in more specific populations and using other types of individual-centered statistical approaches (e.g., latent class modelling). Mose et al. (8) used latent class growth analysis or group-based trajectory modelling (GBTM) to find five 10-year trajectories (2008 to 2017) of musculoskeletal health care use among adult Danes reporting chronic musculoskeletal pain. Kiadaliri et al. (7) identified four trajectories of physical health care consultations within a Swedish osteoarthritis cohort, using GBTM on monthly health care use over the last 12 months of life. As in our study, they both identified low and a high health care utilization groups. However, these two studies modelled the number of health care contacts but failed to analyze the sequence of health care services, or the type of services used (i.e., arthritis related) both considered important factors in defining a care trajectory (31). Overall, our study brings a unique Canadian universal health care perspective, while in line with previous work underlying the presence of heterogeneity in people care utilization, which underlines the importance of using individual-centered statistical approach in arthritis health service research. In other words, depicting health care utilization using single aggregate measures such as average or median number of visits per year is not appropriate to grasp the reality of arthritis patients. The current study is a first step and proof of concept, but further analyses for specific arthritic conditions will be important.

This study showed that participants of the “very high utilization/arthritis-related hospitalizations” group recorded the worst outcomes in terms of pain intensity, pain interference, and self-perceived general and mental health. This was confirmed by multivariable analyses adjusting for comorbidity and other potential confounders. In line with our results, previous studies reported that in the Canadian universal health coverage context, heavy health care use and greater disability are closely linked among patients suffering from chronic non-cancer pain (32) or the general population (33).

In terms of profiles, the “very high utilization/arthritis-related hospitalizations” group had the highest proportion of females, individuals with medical claims related to osteoarthritis, individuals with no high school diploma, and individuals reporting an annual household income below CAN$20,000. Furthermore, it was made up of older individuals and recorded the highest average Charlson Comorbidity Index. Using Andersen's model, Jacobi et al. (34) showed that need factors and predisposing factors explained most of the differences in the use of health care among patients with rheumatoid arthritis. Our results provide valuable information for clinicians and policymakers to identify early on patients who are likely to have a very high health-care utilization and consequently adapt healthcare services (e.g., increase primary care visits to reduce specialist's visits and hospitalization).

It is interesting to point out that the “low utilization” group showed the best outcomes in terms of pain intensity, pain interference, and self-perceived general and mental health. This group also had a higher proportion of males, individuals with a university diploma, reporting an annual household income above CAN$80,000 or more, and were healthier according to the Charlson Comorbidity Index. Those more privileged individuals did not use the health care system as much as the others. Our assessment of outcomes and profile variations between CareT groups can thus be used for better planning of care pathways, but also to raise awareness of the socioeconomic disparities that are present. Our results are aligned with other studies reporting socioeconomic differences in arthritis-related health care (35). Further research should however focus on understanding the presence disparities vs. inequities in arthritis-related health care. Disparities are not undesirable in itself unless it results in some unfairness and injustice, as compared to inequities that are undesirable, should be subject to moral criticism and implies unfairness and injustice (36, 37). Clinicians and policy makers should develop models of care, care pathways and treatment approaches that allow everyone, regardless of socioeconomic status or privilege, to have the chance of experiencing positive health outcomes.

One out of three of our participants perceived their general health as poor or fair. This study suggests that high use of health care services was associated with high pain interference and poor self-perceived health. These results are similar those of Zhao et al. (38), according to which high health care resource utilization is associated with high pain interference in adults living with osteoarthritis in the United States, and urge the need to find solutions to help arthritis patients optimize their health care utilization (for instance, by improving professional practices, including those of nurses, regarding the management of such patients) and decrease patient disability, which in turn could help lead to a decreased utilization of health care services over the next years.

### Additional findings

Our results also show that having osteoarthritis- or other arthritic conditions-related medical claims was associated with a greater risk of seeing some or most activities prevented due to pain or discomfort (Supplementary Digital Content S2). Surprisingly, rheumatoid arthritis medical claims were not associated with pain interference in the multivariable model, although rheumatoid arthritis is known to be debilitating and associated with daily activity limitations (39, 40). In other words, when adjusting for the type of CareT and all relevant potential cofounders, living with rheumatoid arthritis and being supported by health care professionals for its treatment did not appear to be a predictor of greater pain interference. Although those results should be investigated further, a preliminary hypothesis is that patients who use the health care system for rheumatoid arthritis and are cared for with all the recent advances in terms of treatment do not experience severe pain that could interfere with their daily activities. The link between remote region residence and CareTs would also be interesting to investigate further.

### Strengths and limitations

The major strength of this study lies in the use of a very large and rich population survey linked to medical administrative claims. Because arthritis cases were not based on medical claims (rather based on self-reports), we were able to include participants who did not visit the health care system thus providing a greater generalizability of our results to persons living with arthritis in the community. In our sample, the proportion of persons reporting arthritis was 16.3%, which is similar to arthritis prevalence in Canada (2). As state sequence analysis includes the steps of choosing a time unit and prioritization of health events, it cannot be excluded that different time unit/prioritization scheme could have led to different results. As participants were not selected according to administrative data, the index date was defined as CCHS completion and not related to a significant event in the CareTs of arthritis patients (e.g., first diagnosis or hospitalization). Consequently, trajectories modelled in this study represent a random picture of a part of the life course of participants and patterns of health care utilization were quite stable over time. Following this first proof of concept, further studies are needed to better understand CareTs after a first arthritis diagnosis, to conduct a proper sex- and gender-based analysis (SGBA) (41–43) and to focus on arthritic subpopulations (e.g., osteoarthritis, rheumatoid arthritis). In fact, CCHS data do not distinguish among different types of arthritis and only half of participants had in their administrative data a medical claim associated with an arthritic condition diagnosis in the two years prior to CCHS completion. Although the TorSaDE Cohort is generic and does not contain the most commonly used self-reported instruments in the field of pain (44), it still allowed to account for pain characteristics and adjusting for expected confounding variables.

## Conclusion

This study revealed five CareT groups in a wide sample of arthritis patients in Quebec (Canada) and showed that these CareT groups had heterogeneous profiles in terms of sociodemographic characteristics, comorbidity index and health outcomes. Membership to a very high all-cause health care utilization and most frequent arthritis-related hospitalization group was associated with a greater risk of seeing some or most activities prevented by pain or discomfort and higher likelihood of perceiving general health as poor or fair. Besides informing policies for care and public health, these findings could help improve health outcomes for patients with arthritis and, more generally, achieve the common goal of improving population health outcomes by using a more personalised treatment approach based on the CareT types.

## Data Availability

The data analyzed in this study is subject to the following licenses/restrictions: TorSaDE Cohort data links Statistics Canada's Canadian Community Health Survey (CCHS) and Quebec Health Ministry data and are not publicly available. Access has to be granted by the Institut de la statistique du Québec (ISQ) (data holder) and the Commission d’accès à l’information du Québec. Requests to access these datasets should be directed to sad@stat.gouv.qc.ca.

## On behalf of

Past and current members of the TorSaDE Cohort Working Group are as follows: Alain Vanasse (outgoing director), Alexandre Lebel, Amélie Quesnel-Vallée, Anaïs Lacasse (incoming director), André Néron, Anne-Marie Cloutier, Annie Giguère, Benoit Lamarche, Bilkis Vissandjee, Catherine Hudon, Danielle St-Laurent, David Buckeridge, Denis Roy, Geneviève Landry, Gillian Bartlett, Guillaume Blanchet, Hermine Lore Nguena Nguefack, Isabelle Leroux, Jaime Borja, Jean-François Ethier, Josiane Courteau, Lucie Blais, Manon Choinière, Marc Dorais, Marc-André Blanchette, Marc-Antoine Côté-Marcil, Marie-Josée Fleury, Marie-Pascale Pomey, Mike Benigeri, Mireille Courteau, Nadia Sourial, Pasquale Roberge, Pier Tremblay, Pierre Cambon, Roxanne Dault, Sonia Jean, Sonia Quirion, Stéphanie Plante, Thomas Poder, Valérie Émond.
