# Supplementary material for: Distinct care trajectories among persons living with arthritic conditions: A two-year state sequence analysis
Source: Front Pain Res (Lausanne). 2022 Nov 12;3:1014793. doi: 10.3389/fpain.2022.1014793 (PMC9699830; doi:10.3389/fpain.2022.1014793)
Supplement: Supplementary file 1 [file Datasheet1.pdf]

Supplemental digital contents 1: Detailed state sequence analysis

Originally developed for the analysis of protein and DNA sequences [8], state sequence analysis has recently been applied in a variety of contexts, including epidemiology and public health [3,6,10,21]. The main steps applied in this study to compute state sequence analysis are described elsewhere [15] and are similar to those used in other studies conducted by the TorSaDE Cohort Working Group for other ambulatory care sensitive conditions [7,12,14,21,22].

(1) *Cohort of patients, observation period, and time unit:* Among participants who meet the inclusion criteria, health care utilization data was assessed in the two years preceding CCHS completion (index date). Months (4 weeks/28 days periods) were chosen as the time unit (as opposed to days or weeks) to minimize the risk of low number of events per cell and to ensure compliance with ISQ privacy and data release rules. Hence, the care sequence for each participant included 26 potential medical events (one for each time unit of follow-up before the index date).

(2) *Choice and prioritization of medical events (states) of interest:* As more than one medical event may occur during a given month (29.7% of participants had more than one event in at least one of their 26 time units), state sequence analysis requires the prioritization of such events. In other words, in any given 4-month period, should a participant visit more than one type of healthcare setting (e.g., the emergency department (ED) in addition to seeing their general practitioner (GP)), the analysis considers only one of these "events". Medical events (present/absent) were thus prioritized as follows: a) arthritis-related ED visits; b) arthritis-related

hospitalizations; c) pain clinic visits; d) arthritis-related specialist visits; e) arthritis-related GP visits; f) non-arthritis-related contacts with the health care system; and g) no health care visits. Prioritization was established by an expert panel specialized in chronic pain and arthritis health service research, and was guided by multiple factors such as: prioritization of events reflecting lack of access to primary or secondary care (e.g., ED visits were prioritized), the relevance of the events (e.g., hospitalization for arthritis is less likely than ED visits [4,9,20]), the severity of the medical events in order to reflect the possible worsening of the patient's condition, the relative implication of events on healthcare trajectory, and the hierarchy of pain care in the province of Quebec [1]. Pain clinic visits were identified if a claim was associated with a pain clinic establishment code (4X1) or professional activities billed for services rendered in a pain clinic (anesthesia services coded 41055, 41056, 41057, 41058 and 41059). Health care visits were classified as arthritis-related based on the case-finding coding algorithms of the ninth and tenth revisions of the International Classification of Diseases (ICD-9 and ICD-10) for osteoarthritis (715 ICD-9 codes [17,24] and their ICD-10 equivalents), rheumatoid arthritis (714 ICD-9 codes [2,19,23] and their ICD-10 equivalents), and other arthritic conditions (274, 712, 725, 721, 720, 696, 727, 710, 446, 716, 718, 727, 728, 729, 781 ICD-9 codes [16,18,24] and their ICD-10 equivalents).

(3) *Use of an appropriate distance or similarity measure method to calculate the distance between the pairs of sequences, resulting in a distance matrix:* State sequence analysis with Hamming distance, an optimal matching distance algorithm [11,13] implemented in R package TraMineR [5], was used to measure distance or dissimilarities between patient care sequences. This method measures the minimal cost of transforming one sequence into a perfect copy of the other. That minimal cost represents the distance between two

participants' care sequences. Only three kinds of modifications are allowed in optimal matching: substitution, deletion, or insertion [5]. For the primary analysis, a deletion or insertion cost of 1 was chosen and a substitution-cost matrix based on the estimated transition rates. The rationale was to set a high cost when changes between two medical events were rarely observed, and a lower cost when they were frequent [5]. A distance matrix was created at the end of this step.

(4) *Selection and application of a classification method based on the calculated distance matrix that results in distinct groups of patients sharing similar patterns of health care utilization (CareT groups):* Based on the distance matrix created in step 3, a hierarchical cluster analysis approach was used to create groups of participants with similar care sequences (CareT groups). The optimal number of CareT groups was chosen based on their clinical relevance, interpretability, size (at least 5% of the sample in each CareT group), and analytical tools (dendrogram and inertia jump curve which indicate the extent of information gained with each added CareT group) [7,21].

(5) *Visual representation:* Chronograms (state distribution plots), showing the distribution of medical events for each time unit point, were created and used to display CareT groups (one chronogram per group) and narrative description (label) was assigned to each CareT. In chronograms to be published, time points with fewer than 15 participants had to be hidden to ensure compliance with ISQ privacy and data release rules. That said, data description in this paper reflects entirely our interpretation of the original graphs. State sequence analysis was applied to the whole sample.

References

[1] AETMIS. Prise en charge de la douleur chronique (non cancéreuse) : Organisation des services de santé. Montréal (Qc): Agence d'évaluation des technologies et des modes d'intervention en santé, 2006.

[2] Chung CP, Rohan P, Krishnaswami S, McPheeters ML. A systematic review of validated methods for identifying patients with rheumatoid arthritis using administrative or claims data. Vaccine 2013;31:K41-K61.

[3] Defossez G, Rollet A, Dameron O, Ingrand P. Temporal representation of care trajectories of cancer patients using data from a regional information system: an application in breast cancer. BMC Medical Informatics and Decision Making 2014;14(24).

[4] Doherty E, O'Neill C. Estimating the health-care usage associated with osteoarthritis and rheumatoid arthritis in an older adult population in Ireland. Journal of Public Health 2014;36(3):504-510.

[5] Gabadinho A, Ritschard G, Müller NS, Studer M. Analyzing and Visualizing State Sequences in R with TraMineR. Journal of Statistical Software 2011;40(4):1-37.

[6] Haenssgen MJ, Ariana P. Healthcare access: A sequence-sensitive approach. SSM Popul Health 2017;3:37-47.

[7] Henri S, Herrera R, Vanasse A, Forget A, Blais L. Trajectories of care in patients with chronic obstructive pulmonary disease: A sequence analysis. Canadian Journal of Respiratory, Critical Care, and Sleep Medicine 2021:1-11.

[8] Karlin S, Ghandour G. Comparative statistics for DNA and protein sequences: multiple sequence analysis. Proc Natl Acad Sci U S A 1985;82(18):6186-6190.

[9] Lacasse A, Bourgault P, Choinière M. Fibromyalgia-related costs and loss of productivity: a substantial societal burden. BMC Musculoskeletal Disorders 2016;17(1):168.

[10] Le Meur N, Gao F, Bayat S. Mining care trajectories using health administrative information systems: the use of state sequence analysis to assess disparities in prenatal care consumption. BMC Health Services Research 2015;15(1):200.

[11] Lesnard L, de Saint Pol T. Introduction aux méthodes d'appariement optimal (Optimal Matching Analysis). Bulletin de méthodologie sociologique/Bulletin of sociological methodology 2006;90.

[12] Letarte L, Gagnon P, McKay R, Quesnel-Vallée A, Waygood E, Lebel A, Group TCW. Examining longitudinal patterns of individual neighborhood deprivation trajectories in the province of Quebec: A sequence analysis application. Social Science & Medicine 2021:113695.

[13] Martin TF. Advancing Dynamic Family Theories: Applying Optimal Matching Analysis to Family Research. Journal of Family Theory & Review 2015;7(4):482-497.

[14] McKay R, Letarte L, Lebel A, Quesnel-Vallée A. Exploring social inequalities in healthcare trajectories following diagnosis of diabetes: a state sequence analysis of linked survey and administrative data. BMC Health Serv Res 2022;22(1):131.

[15] Nguefack HLN, Pagé MG, Katz J, Choinière M, Vanasse A, Dorais M, Samb OM, Lacasse A. Trajectory modelling techniques useful to epidemiological research: a comparative narrative review of approaches. Clinical Epidemiology 2020;12:1205.

[16] Rector TS, Wickstrom SL, Shah M, Thomas Greeenlee N, Rheault P, Rogowski J, Freedman V, Adams J, Escarce JJ. Specificity and sensitivity of claims-based algorithms for identifying members of Medicare+Choice health plans that have chronic medical conditions. Health Serv Res 2004;39(6 Pt 1):1839-1857.
[17] Shrestha S, Dave AJ, Losina E, Katz JN. Diagnostic accuracy of administrative data algorithms in the diagnosis of osteoarthritis: a systematic review. BMC medical informatics and decision making 2016;16(1):1-12.
[18] Singh JA, Holmgren AR, Noorbaloochi S. Accuracy of Veterans Administration databases for a diagnosis of rheumatoid arthritis. Arthritis Rheum 2004;51(6):952-957.
[19] Slim ZF, de Moura CS, Bernatsky S, Rahme E. Identifying rheumatoid arthritis cases within the Quebec Health Administrative Database. The Journal of rheumatology 2019;46(12):1570-1576.
[20] Son KM, Kim J-R, Park HA, Kim HA. Trends in hospital visits and healthcare costs of gout and seropositive rheumatoid arthritis in Korea from 2010 to 2017 using National Healthcare Claims. The Korean Journal of Internal Medicine 2022;37(3):681.
[21] Vanasse A, Courteau J, Courteau M, Benigeri M, Chiu YM, Dufour I, Couillard S, Larivée P, Hudon C. Healthcare utilization after a first hospitalization for COPD: a new approach of State Sequence Analysis based on the'6W'multidimensional model of care trajectories. BMC Health Services Research 2020;20(1):1-15.
[22] Vanasse A, Courteau J, Courteau M, Roy M-A, Stip E, Fleury M-J, Lesage A, Brodeur S. Multidimensional analysis of adult patients' care trajectories before a first diagnosis of schizophrenia. Schizophrenia 2022;8(1):52.
[23] Widdifield J, Bernatsky S, Paterson JM, Tu K, Ng R, Thorne JC, Pope JE, Bombardier C. Accuracy of Canadian health administrative databases in identifying patients with rheumatoid arthritis: a validation study using the medical records of rheumatologists. Arthritis care & research 2013;65(10):1582-1591.
[24] Widdifield J, Labrecque J, Lix L, Paterson JM, Bernatsky S, Tu K, Ivers N, Bombardier C. Systematic review and critical appraisal of validation studies to identify rheumatic diseases in health administrative databases. Arthritis care & research 2013;65(9):1490-1503.

Supplemental digital content 2: Multivariable logistic regression model exploring associations between CT groups' membership and pain interference

Participants characteristics	Adjusted OR	95% Confidence interval		p-value
**Care trajectories**				
Care trajectories membership (vs Low utilization group)				
High utilization/specialists	1.138	0.934	1.388	0.2001
Moderate utilization/ED	0.978	0.804	1.191	0.8272
Very high utilization/arthritis-related hospitalizations	**1.432**	**1.132**	**1.811**	**0.0027**
Moderate utilization/arthritis-related physicians	0.999	0.820	1.216	0.9919
**Sociodemographic profile**				
Age (years)	**0.991**	**0.985**	**0.997**	**0.0050**
Sex (women vs. men)	**0.863**	**0.745**	**0.999**	**0.0484**
Race/ethnicity (Non-Caucasian vs. Caucasian)	1.324	0.760	2.306	0.3214
Indigenous (Yes vs No)	0.979	0.503	1.908	0.9513
Country of birth (Canada vs. other)	**0.685**	**0.471**	**0.996**	**0.0474**
Education level (vs. No secondary education diploma)				
Secondary education diploma	1.167	0.949	1.434	0.1428
Post-secondary education diploma	**1.245**	**1.062**	**1.459**	**0.0069**
University education diploma	1.255	0.988	1.594	0.0623

Marital status (In a relationship vs. Not in a relationship)	0.908	0.778	1.059	0.2172
Household income ($) (vs. < 20 000)				
20 000 -39 999	0.912	0.769	1.082	0.2904
40 000 - 59 999	0.863	0.688	1.084	0.2048
60 000 -79 999	0.901	0.677	1.200	0.4759
≥ 80 000	0.836	0.616	1.135	0.2501
Region of residence (Remote region vs. Non-remote region)	0.953	0.818	1.110	0.5351
Geographic area (Rural vs. Urban)	1.064	0.920	1.231	0.4045
CCHS cycle (vs 2007-2008)				
2009-2010	0.978	0.813	1.176	0.8129
2011-2012	**1.434**	**1.199**	**1.714**	**<.0001**
2013-2014	1.015	0.841	1.224	0.8797
Drug insurance status (Covered vs. Not covered)	1.092	0.918	1.300	0.3204
**Healthcare**				
Access to a pain clinic (Yes vs. No)	1.135	0.893	1.442	0.3015
Access to a family physician (Yes vs. No)	1.037	0.832	1.293	0.7446
Number of different prescribers in the past 12 months	1.011	0.994	1.028	0.2056
RA medical claims (Yes vs. No)	1.118	0.875	1.427	0.3731
OA medical claims (Yes vs. No)	**1.383**	**1.204**	**1.590**	**<.0001**
Medical claims related to other arthritis conditions except RA and OA (Yes vs. No)	**1.306**	**1.142**	**1.493**	**<.0001**

**Pain symptoms**				
Self-reported pain symptoms -Pain intensity (Moderate/Severe vs. None/Mild)	**24.776**	**19.868**	**30.898**	**<.0001**
Back pain, except fibromyalgia and arthritis (Yes vs. No)	**1.453**	**1.275**	**1.655**	**<.0001**
**General health profile**				
Charlson Comorbidity Index in the past 12 months	1.000	0.956	1.047	0.9876
Perceived general health (Fair/Poor vs. Excellent/very good/good)	**3.250**	**2.825**	**3.738**	**<.0001**
Perceived mental health Fair/Poor vs. Excellent/very good/good)	1.187	0.965	1.459	0.1039
Lifestyle habits-Kind of alcohol consumption in the past 12 months (vs. Regular)				
Occasional	0.865	0.738	1.015	0.0758
Has not drunk	0.921	0.767	1.105	0.3736
Lifestyle habits-Kind of smoker (vs. Regular)				
Occasional	**1.263**	**1.067**	**1.495**	**0.0066**
Never	0.897	0.599	1.345	0.6002
Body mass index (vs. Normal weight)				
Underweight	**1.719**	**1.168**	**2.531**	**0.0060**
Overweight	**1.183**	**1.009**	**1.386**	**0.0379**
Obese class 1	**1.491**	**1.235**	**1.800**	**<.0001**
Obese class 2	**1.787**	**1.368**	**2.335**	**<.0001**
Obese class 3	**2.098**	**1.560**	**2.821**	**<.0001**

Table footnotes: P-values <.05 are reported in bold

Supplemental digital content 3: Multivariable logistic regression model exploring associations between CT groups' membership and perceived general health

Participants characteristics	Adjusted OR	95% Confidence interval		p-value
**Care trajectories**				
Care trajectories membership (vs Low utilization group)				
High utilization/specialists	**1.553**	**1.321**	**1.826**	**<.0001**
Moderate utilization/ED	**1.247**	**1.064**	**1.460**	**0.0063**
Very high utilization/arthritis-related hospitalizations	**1.783**	**1.461**	**2.177**	**<.0001**
Moderate utilization/arthritis-related physicians	**1.483**	**1.266**	**1.737**	**<.0001**
**Sociodemographic profile**				
Age (years)	1.003	0.998	1.008	0.2583
Sex (women vs. men)	**0.636**	**0.565**	**0.715**	**<.0001**
Race/ethnicity (Non-Caucasian vs. Caucasian)	1.343	0.825	2.186	0.2362
Indigenous (Yes vs No)	0.725	0.403	1.306	0.2840
Country of birth (Canada vs. other)	0.799	0.593	1.075	0.1380
Education level (vs. No secondary education diploma)				
Secondary education diploma	**0.671**	**0.566**	**0.796**	**<.0001**
Post-secondary education diploma	**0.644**	**0.566**	**0.733**	**<.0001**
University education diploma	**0.491**	**0.401**	**0.603**	**<.0001**

Marital status (In a relationship vs. Not in a relationship)	1.124	0.992	1.274	0.0670
Household income ($) (vs. < 20 000)				
20 000 -39 999	**0.702**	**0.612**	**0.805**	**<.0001**
40 000 - 59 999	**0.496**	**0.411**	**0.597**	**<.0001**
60 000 -79 999	**0.546**	**0.431**	**0.692**	**<.0001**
≥ 80 000	**0.436**	**0.337**	**0.562**	**<.0001**
Region of residence (Non-remote region vs. Remote region)	1.088	0.960	1.232	0.1858
Geographic area (Rural vs. Urban)	1.116	0.991	1.257	0.0693
CCHS cycle (vs 2007-2008)				
2009-2010	0.977	0.845	1.129	0.7492
2011-2012	**0.742**	**0.641**	**0.858**	**<.0001**
2013-2014	**0.826**	**0.710**	**0.961**	**0.0132**
Drug insurance status (Covered vs. Not covered)	**1.180**	**1.020**	**1.365**	**0.0258**
**Healthcare**				
Access to a pain clinic (Yes vs. No)	**1.311**	**1.065**	**1.612**	**0.0105**
Access to a family physician (Yes vs. No)	0.912	0.765	1.086	0.2999
Number of different prescribers in the past 12 months	**1.034**	**1.020**	**1.049**	**<.0001**
RA medical claims (Yes vs. No)	**1.550**	**1.265**	**1.899**	**<.0001**
OA medical claims (Yes vs. No)	0.974	0.866	1.094	0.6534
Medical claims related to other arthritis conditions except RA and OA (Yes vs. No)	**1.198**	**1.073**	**1.338**	**0.0013**

**Pain symptoms**				
Self-reported pain symptoms -Pain intensity (Moderate/Severe vs. None/Mild)	**1.946**	**1.728**	**2.192**	**<.0001**
Self-reported pain symptoms -Pain interference (Some/Most vs. None/A few)	**3.141**	**2.728**	**3.616**	**<.0001**
Back pain, except fibromyalgia and arthritis (Yes vs. No)	**1.531**	**1.372**	**1.709**	**<.0001**
**General health profile**				
Charlson Comorbidity Index in the past 12 months	**1.116**	**1.075**	**1.159**	**<.0001**
Perceived mental health Fair/Poor vs. Excellent/very good/good)	**7.185**	**5.806**	**8.892**	**<.0001**
Lifestyle habits-Kind of alcohol consumption in the past 12 months (vs. Regular)				
Occasional	**0.484**	**0.427**	**0.550**	**<.0001**
Has not drunk	**0.598**	**0.515**	**0.693**	**<.0001**
Lifestyle habits-Kind of smoker (vs. Regular)				
Occasional	**1.631**	**1.413**	**1.883**	**<.0001**
Never	1.364	0.976	1.907	0.0693
Body mass index (vs. Normal weight)				
Underweight	**1.429**	**1.031**	**1.980**	**0.0319**
Overweight	**1.138**	**1.003**	**1.291**	**0.0446**
Obese class 1	**1.377**	**1.179**	**1.608**	**<.0001**
Obese class 2	**1.860**	**1.471**	**2.352**	**<.0001**
Obese class 3	**2.087**	**1.605**	**2.714**	**<.0001**

Table footnotes: P-values <.05 are reported in bold

Supplemental digital content 4: Multivariable logistic regression model exploring associations between CT groups' membership and pain interference using multiple imputation data

Participants characteristics	Adjusted OR	95% Confidence interval		p-value
**Care trajectories**				
Care trajectories membership (vs Low utilization group)				
High utilization/specialists	1.038	0.870	1.238	0.6820
Moderate utilization/ED	0.968	0.818	1.145	0.7035
Very high utilization/arthritis-related hospitalizations	**1.340**	**1.108**	**1.620**	**0.0026**
Moderate utilization/arthritis-related physicians	0.972	0.798	1.183	0.7764
**Sociodemographic profile**				
Age (years)	0.995	0.989	1.002	0.1492
Sex (women vs. men)	**0.864**	**0.755**	**0.988**	**0.0402**
Race/ethnicity (Non-Caucasian vs. Caucasian)	1.030	0.705	1.506	0.8780
Indigenous (Yes vs No)	1.223	0.7036	2.127	0.4814
Country of birth (Canada vs. other)	0.809	0.642	1.020	0.0740
Education level (vs. No secondary education diploma)				
Secondary education diploma	**1.219**	**1.005**	**1.478**	**0.0529**
Post-secondary education diploma	**1.196**	**1.039**	**1.376**	**0.0145**
University education diploma	1.170	0.936	1.462	0.1781

Marital status (In a relationship vs. Not in a relationship)	0.897	0.779	1.034	0.1408
Household income ($) (vs. < 20 000)				
20 000 -39 999	0.946	0.813	1.101	0.4750
40 000 - 59 999	0.856	0.670	1.094	0.2314
60 000 -79 999	0.899	0.702	1.150	0.3992
≥ 80 000	0.945	0.697	1.281	0.7187
Region of residence (Remote region vs. Non-remote region)	0.963	0.812	1.142	0.6714
Geographic area (Rural vs. Urban)	1.066	0.904	1.255	0.4598
CCHS cycle (vs 2007-2008)				
2009-2010	0.987	0.832	1.170	0.8763
2011-2012	**1.281**	**1.090**	**1.506**	**0.0027**
2013-2014	0.936	0.791	1.108	0.4423
2015-2016	0.399	0.025	6.346	0.5487
Drug insurance status (Covered vs. Not covered)	1.077	0.897	1.293	0.4385
**Healthcare**				
Access to a pain clinic (Yes vs. No)	1.101	0.880	1.376	0.4058
Access to a family physician (Yes vs. No)	1.040	0.885	1.223	0.6321
Number of different prescribers in the past 12 months	1.013	0.997	1.028	0.1251
RA medical claims (Yes vs. No)	1.146	0.912	1.439	0.2463
OA medical claims (Yes vs. No)	**1.267**	**1.098**	**1.462**	**0.0036**
Medical claims related to other arthritis conditions except RA and OA (Yes vs. No)	**1.253**	**1.091**	**1.439**	**0.0045**

**Pain symptoms**				
Self-reported pain symptoms -Pain intensity (Moderate/Severe vs. None/Mild)	**24.328**	**17.866**	**33.128**	**<.0001**
Back pain, except fibromyalgia and arthritis (Yes vs. No)	**1.440**	**1.270**	**1.633**	**<.0001**
**General health profile**				
Charlson Comorbidity Index in the past 12 months	0.999	0.956	1.045	0.9710
Perceived general health (Fair/Poor vs. Excellent/very good/good)	**3.129**	**2.595**	**3.773**	**<.0001**
Perceived mental health Fair/Poor vs. Excellent/very good/good)	1.287	0.987	1.679	0.0923
Lifestyle habits-Kind of alcohol consumption in the past 12 months (vs. Regular)				
Occasional	**0.827**	**0.715**	**0.957**	**0.0141**
Has not drunk	0.868	0.738	1.021	0.0924
Lifestyle habits-Kind of smoker (vs. Regular)				
Occasional	**1.316**	**1.113**	**1.555**	**0.0034**
Never	0.950	0.658	1.372	0.7871
Body mass index (vs. Normal weight)				
Underweight	**1.531**	**1.058**	**2.214**	**0.0276**
Overweight	**1.159**	**0.973**	**1.381**	**0.1189**
Obese class 1	**1.477**	**1.266**	**1.723**	**<.0001**
Obese class 2	**1.787**	**1.370**	**2.331**	**0.0003**
Obese class 3	**1.871**	**1.382**	**2.532**	**0.0005**

Table footnotes: P-values <.05 are reported in bold

Supplemental digital content 5: Multivariable logistic regression model exploring associations between CT groups' membership and perceived general health using multiple imputation data

Participants characteristics	Adjusted OR	95% Confidence interval		p-value
**Care trajectories**				
Care trajectories membership (vs Low utilization group)				
High utilization/specialists	**1.464**	**1.288**	**1.664**	**<.0001**
Moderate utilization/ED	**1.178**	**1.039**	**1.335**	**0.0096**
Very high utilization/arthritis-related hospitalizations	**1.789**	**1.525**	**2.098**	**<.0001**
Moderate utilization/arthritis-related physicians	**1.362**	**1.201**	**1.545**	**<.0001**
**Sociodemographic profile**				
Age (years)	**1.005**	**1.001**	**1.009**	**0.0205**
Sex (women vs. men)	**0.712**	**0.648**	**0.782**	**<.0001**
Race/ethnicity (Non-Caucasian vs. Caucasian)	1.226	0.869	1.730	0.2240
Indigenous (Yes vs No)	0.883	0.565	1.381	0.5490
Country of birth (Canada vs. other)	**0.803**	**0.665**	**0.970**	**0.0260**
Education level (vs. No secondary education diploma)				
Secondary education diploma	**0.657**	**0.569**	**0.746**	**<.0001**
Post-secondary education diploma	**0.665**	**0.595**	**0.744**	**<.0001**
University education diploma	**0.495**	**0.420**	**0.583**	**<.0001**
Marital status (In a relationship vs. Not in a relationship)	**1.147**	**1.040**	**1.266**	**0.0061**

Household income ($) (vs. < 20 000)				
20 000 -39 999	**0.741**	**0.662**	**0.830**	**<.0001**
40 000 - 59 999	**0.545**	**0.470**	**0.633**	**<.0001**
60 000 -79 999	**0.506**	**0.419**	**0.610**	**<.0001**
≥ 80 000	**0.422**	**0.345**	**0.516**	**<.0001**
Region of residence (Remote region vs. Non-remote region)	1.090	0.987	1.205	0.0798
Geographic area (Rural vs. Urban)	1.091	0.990	1.202	0.0840
CCHS cycle (vs 2007-2008)				
2009-2010	0.965	0.843	1.105	0.6369
2011-2012	**0.755**	**0.661**	**0.862**	**<.0001**
2013-2014	**0.832**	**0.725**	**0.954**	**0.0103**
2015-2016	0.624	0.234	1.6654	0.3658
Drug insurance status (Covered vs. Not covered)	**1.195**	**1.065**	**1.342**	**0.0029**
**Healthcare**				
Access to a pain clinic (Yes vs. No)	**1.364**	**1.155**	**1.611**	**0.0003**
Access to a family physician (Yes vs. No)	0.995	0.873	1.134	0.9429
Number of different prescribers in the past 12 months	**1.033**	**1.022**	**1.045**	**<.0001**
RA medical claims (Yes vs. No)	**1.520**	**1.285**	**1.798**	**<.0001**
OA medical claims (Yes vs. No)	0.917	0.833	1.010	0.0812
Medical claims related to other arthritis conditions except RA and OA (Yes vs. No)	**1.286**	**1.174**	**1.409**	**<.0001**

**Pain symptoms**				
Self-reported pain symptoms -Pain intensity (Moderate/Severe vs. None/Mild)	**1.438**	**1.195**	**1.732**	**<.0001**
Self-reported pain symptoms -Pain interference (Some/Most vs. None/A few)	**2.983**	**2.496**	**3.565**	**<.0001**
Back pain, except fibromyalgia and arthritis (Yes vs. No)	**1.6492**	**1.494**	**1.821**	**<.0001**
**General health profile**				
Charlson Comorbidity Index in the past 12 months	**1.120**	**1.087**	**1.154**	**<.0001**
Perceived mental health Fair/Poor vs. Excellent/very good/good)	**7.575**	**6.332**	**9.062**	**<.0001**
Lifestyle habits-Kind of alcohol consumption in the past 12 months (vs. Regular)				
Occasional	**0.508**	**0.459**	**0.563**	**<.0001**
Has not drunk	**0.658**	**0.584**	**0.741**	**<.0001**
Lifestyle habits-Kind of smoker (vs. Regular)				
Occasional	**1.684**	**1.487**	**1.907**	**<.0001**
Never	1.246	0.957	1.623	0.1111
Body mass index (vs. Normal weight)				
Underweight	1.307	0.995	1.716	0.0524
Overweight	**1.108**	**0.998**	**1.229**	**0.0554**
Obese class 1	**1.364**	**1.202**	**1.548**	**<.0001**
Obese class 2	**1.771**	**1.430**	**2.193**	**<.0001**
Obese class 3	**2.062**	**1.658**	**2.565**	**<.0001**

Table footnotes: P-values <.05 are reported in bold
